# Single-inspiratory quantitative CT nomogram for enhanced PRISm and COPD differentiation: a cross-sectional study with interpretable diagnostic boundaries

**DOI:** 10.7717/peerj.20575

**Published:** 2026-01-14

**Authors:** Qi Dai, Xiaoxiao Zhu, Qifeng Hua, Jingfeng Zhang, Zhaoxing Dong, Jianjun Zheng, Jingyun Shi

**Affiliations:** 1 School of Medicine, Tongji University, Shanghai, China; 2 Department of Radiology, Ningbo No. 2 Hospital, Ningbo, China; 3 Department of Respiratory and Critical Care Medicine, Ningbo No. 2 Hospital, Ningbo, China; 4 Department of Radiology, Shanghai Pulmonary Hospital, School of Medicine, Tongji University, Shanghai, China

**Keywords:** PRISm, COPD, Single-inspiratory CT, Nomogram, Early detection

## Abstract

**Background:**

Differentiating preserved ratio impaired spirometry (PRISm) from chronic obstructive pulmonary disease (COPD) is challenging. Traditional biphasic CT scans are limited by radiation exposure, while single-inspiratory CT-based deep learning lacks interpretability. This study aimed to develop a single-inspiratory quantitative computed tomography (QCT) nomogram integrating parenchymal, airway, and vascular parameters to redefine imaging definition boundaries.

**Methods:**

This retrospective study (approved by Ethics Committee YJ-NBEY-KY-2023-107-01) screened 1,265 patients from Ningbo No. 2 Hospital (January 2021–December 2023), yielding 658 eligible participants (Normal: 135, PRISm: 328, COPD: 195) based on predefined inclusion/exclusion criteria. Single-inspiratory CT metrics (parenchymal, airway, vascular) were quantified using the Aview® system. Four logistic regression models distinguished PRISm from normal and COPD group receiver operating characteristic-area under the curve (ROC-AUC) evaluated performance.

**Results:**

Progressive deterioration in age (COPD: 73.3 *vs*. PRISm: 69.1 *vs*. Normal: 64.1 years), male predominance (84.6% COPD *vs*. 57.9% PRISm), pulmonary function (FEV1%, FEV1/FVC), and CT markers (Pi10: PRISm 3.65 *vs*. Normal 3.26, *P* < 0.001) were observed. PRISm showed reduced superficial vessel diameter (AVD9: 2.64 mm *vs*. Normal 2.95 mm, *P* < 0.001). Diagnostic models achieved AUCs up to 0.984 (PRISm *vs*. severe COPD) and 0.853 (PRISm *vs*. all COPD).

**Conclusion:**

The QCT nomogram robustly differentiates PRISm from COPD, highlighting reduced superficial vessel diameter as a key biomarker. This radiation-efficient approach enables early COPD stratification *via* interpretable structural-functional metrics.

## Introduction

Chronic obstructive pulmonary disease (COPD) stands as a leading cause of mortality globally, characterized by persistent airflow limitation and chronic inflammation. According to modeling estimates from the Global Burden of Disease database (Boers et al., 2023), the number of COPD cases among individuals aged 25 years and older is projected to increase by 23% between 2020 and 2050, reaching nearly 600 million patients worldwide by 2050. The disease burden is escalating rapidly, particularly in low- and middle-income countries. Compared to 1990, China witnessed a 66.20% increase in new COPD cases and a 66.76% rise in overall prevalence by 2019 (Li et al., 2023). Recent research by Li et al. (2024) highlights that the pathogenesis of COPD is highly complex and heterogeneous, involving multiple inflammatory mechanisms that collectively drive disease progression. Despite this complexity and advancements in understanding its pathophysiology, COPD remains a preventable, diagnosable, and manageable condition, with early intervention shown to significantly improve lung function and prognosis (Dharmage et al., 2023).

However, current diagnostic approaches for COPD heavily rely on spirometry post-bronchodilator, defining airflow obstruction as a ratio of forced expiratory volume in one second to forced vital capacity (FEV1/FVC) < 0.70 (Venkatesan, 2025). Spirometry has significant limitations: it can only detect abnormalities after substantial structural damage (approximately 30% loss of lung tissue) and fails to differentiate subtypes with overlapping phenotypes, such as preserved ratio impaired spirometry (PRISm). PRISm is defined as FEV1 <80% predicted and FEV1/FVC ≥0.70, affecting 7–20% of the global population and increasingly recognized as a precursor to COPD progression (Wijnant et al., 2020; Kaise et al., 2021; Washio et al., 2022). Nonetheless, PRISm is excluded from traditional COPD diagnostic frameworks, leaving these patients underserved in clinical practice (Huang et al., 2024). Epidemiological data from the COPDGene cohort indicate that 32.6% of PRISm patients progress to COPD within 4–5 years, accompanied by an increased risk of cardiovascular death and exacerbations (Young et al., 2019).

Quantitative computed tomography (QCT) may emerged as a powerful tool to address these gaps. High-resolution CT (HRCT) is capable of precisely quantifying parenchymal destruction, airway remodeling, and vascular changes—features that are integral components of the pathophysiology in COPD and PRISm (Wei et al., 2018). Lu et al. (2022) demonstrated that PRISm patients exhibit distinct radiological features, the emphasis was placed on the potential of parametric response mapping (PRM), which can effectively differentiate between normal and PRISm (area under the curve (AUC) = 0.786). However, existing protocols typically require biphasic (inspiratory/expiratory) CT scans to assess small airway dysfunction, doubling radiation exposure and thereby limiting clinical feasibility.

Despite these findings, radiomics and deep learning (DL) have emerged as promising tools for the non-invasive assessment of lung diseases, including PRISm. Radiomics involves the extraction of high-dimensional quantitative features from medical images, which can be used to characterize tissue heterogeneity and predict disease outcomes, a comprehensive model combining deep learning, radiomics features, and questionnaire data achieved the highest diagnostic performance (AUC = 0.971) (Zhu et al., 2024). Deep learning, particularly convolutional neural networks (CNNs), has shown great potential in automating the segmentation of lung regions and extracting meaningful features from CT images. In a multicenter retrospective study, the integrated Rad-score and clinical model demonstrated enhanced diagnostic accuracy for distinguishing PRISm from COPD, with AUCs of 0.82, 0.77, and 0.841 across the respective datasets (Zhou et al., 2024b). Similarly, Zhou et al. (2024a) used DL-based segmentation to extract radiomics features from whole-lung CT images, achieving significant improvements in the prediction of PRISm, the CT-based whole lung radiomics nomogram, incorporating 14 stable features along with age, body mass index (BMI), and gender, demonstrated superior performance in identifying PRISm from non-COPD subjects compared to clinical or radiomics models alone, achieving AUCs of 0.787. These technologies offer several advantages over traditional methods, including the ability to analyze large datasets, detect subtle changes in lung structure, and provide quantitative metrics that are not visible to the human eye (Wu et al., 2024).

However, the application of radiomics and DL in PRISm diagnosis is not without challenges. One major limitation is the need for large, well-annotated datasets to train robust models. Additionally, the reproducibility of radiomics features is significantly impacted by inter-institutional variations in CT acquisition parameters, such as slice thickness and reconstruction algorithms, which can result in a 30–50% decrease in feature stability (Pfaehler et al., 2021). Lastly, the “black-box” nature of DL models poses a substantial barrier to clinical adoption, as it hinders clinicians from making causal inferences about key biomarkers that are critical for understanding disease mechanisms and guiding therapeutic decisions.

Given these advancements, our study aims to develop a quantitative CT-based model for the early diagnosis of PRISm. QCT offers multiple clinical advantages: (1) early detection of structural abnormalities before spirometric decline, (2) objective differentiation between PRISm and COPD phenotypes, (3) risk stratification for disease progression, (4) guidance for personalized therapeutic interventions, and (5) precise lung densitometry for monitoring treatment response (Zhang et al., 2023; Lukhumaidze et al., 2025). By analyzing the radiomics features of lung parenchyma, bronchi, and pulmonary vasculature, we seek to identify distinct imaging biomarkers that can differentiate PRISm from COPD and normal spirometry. This approach not only reduces the radiation exposure associated with biphasic CT scans but also provides a more comprehensive and objective assessment of lung function, which is crucial for the early detection and management of PRISm.

## Materials and Methods

This study was reviewed and approved by the Ethics Committee of the Ningbo No. 2 Hospital (Approval Number: YJ-NBEY-KY-2023-107-01), in accordance with the ethical standards set forth in the Declaration of Helsinki for retrospective studies. Considering that the research entailed a secondary analysis of de-identified clinical data and did not involve any intervention in the clinical treatment processes, the Ethics Committee granted a waiver for obtaining patients’ informed consent.

### Research design and participants

A single-center retrospective cohort design was used to enroll 1,265 patients who underwent deep inspiratory chest CT scans and standardized Pulmonary Function Tests (PFTs) at the respiratory department outpatient clinic of the Ningbo No. 2 Hospital between January 2021 and December 2023. Inclusion criteria were: age ≥40 years with complete clinical data (including smoking index and occupational exposure history), a time interval ≤30 days between CT and PFTs examinations (median interval of 7 days, interquartile range of 3–16 days), and CT image quality meeting the requirements for radiomics analysis (respiratory motion artifact score ≤2 on a five-level Likert scale). Exclusion criteria included: acute respiratory events within 3 months prior to enrollment (COPD exacerbation, pneumonia, or pulmonary embolism), active pulmonary tumors or metastatic lesions, significant structural abnormalities (extensive lung collapse, pleural effusion >500 ml, or usual interstitial pneumonia (UIP)-pattern pulmonary fibrosis), and artifacts in CT images that could affect image analysis, such as the presence of metal implants in the lungs.

Participants were categorized into three groups based on the Global Initiative for Chronic Obstructive Lung Disease (GOLD) guidelines: (1) Normal Spirometry Group (*n* = 135): FEV1/FVC ≥ 0.70 and FEV1 ≥ 80% of predicted value, with no chronic respiratory symptoms (mMRC score ≤ 1); (2) PRISm Group (*n* = 328): FEV1/FVC ≥ 0.70 but FEV1 < 80% of predicted value, excluding other causes of restrictive ventilatory defects; (3) COPD Group (*n* = 195), further classified according to the degree of airflow limitation into GOLD 1 (FEV1 ≥ 80%, *n* = 33), GOLD 2 (50% ≤ FEV1 < 80%, *n* = 93), GOLD 3 (30% ≤ FEV1 < 50%, *n* = 55), and GOLD 4 (FEV1 < 30% or combined with type II respiratory failure, *n* = 14).

**Figure 1:**
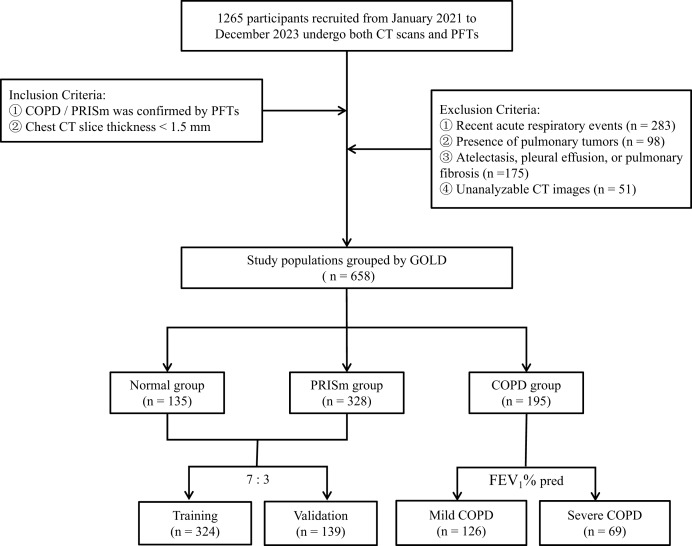
Flowchart of research object selection and grouping. PFTs Pulmonary Function Tests, GOLD Global Initiative for Chronic Obstructive Pulmonary Disease.

A random sampling strategy, executed with R 4.4.2 (downloaded from the official website http://www.R-project.org), was used to divide the dataset, ensuring each subset is representative. The PRISm group (*n* = 328) and the normal group (*n* = 135) were allocated to the training set (*n* = 324) and internal validation set (*n* = 139) at a ratio of 7:3, with baseline characteristics confirmed to be balanced using the Kolmogorov-Smirnov test (*P* > 0.05). Based on the predicted FEV1 values, the COPD group was divided into two subgroups: mild (GOLD 1–2, *n* = 126) and severe (GOLD 3–4, *n* = 69). The screening and division process for all study participants is illustrated in Fig. 1.

### Pulmonary function and imaging data acquisition

PFTs were conducted using the GANSHORN PowerCube Diffusion+ system (Ganshorn Medizin Electronic GmbH, Germany), strictly adhering to the American Thoracic Society/European Respiratory Society (ATS/ERS) 2019 joint guidelines (Graham et al., 2019). The mean of the best three post-bronchodilator measurements was recorded to obtain airflow dynamics indices (FEV1, FVC, FEV1/FVC, peak expiratory flow (PEF), mid-expiratory flow (MEF25-75)), lung volume parameters (total lung capacity (TLC) measured by plethysmography, residual volume (RV) determined by nitrogen washout), and diffusion capacity (Diffusing Capacity of the Lungs for Carbon Monoxide-Single Breath (DLCO-SB) assessed by single-breath method, corrected for hemoglobin).

Chest CT scans were performed on three Siemens systems (SOMATOM Definition AS+, go Top, Force) with specific parameters including tube voltages of 120/100/90–110 kV, tube currents of 100/75–165/50–80 mAs, slice thicknesses of 0.6–1.0 mm, and reconstruction algorithms B80f, I70f, Qr40. Image preprocessing involved non-local means filtering (σ = 2.0), B-spline elastic registration (Elastix v5.0), and Hounsfield unit (HU) standardization (air: −1,000 HU, water: 0 HU).

Quantitative analysis of pulmonary CT images was performed using the Aview® system (Coreline Soft Inc., Seoul, South Korea). Lung parenchymal features—mean lung density (MLD), emphysema index (EI), and pixel indices PI-1/PI-15—were determined through histogram thresholding. The areas of low attenuation regions located 9, 15, and 21 mm from the pleura (Surface Low Attenuation Area, S_LAA_) were calculated. Airway parameters, including square root of wall area of a hypothetical airway with an internal perimeter of 10 mm (Pi10) and wall area percentage (WA%) of sixth grade bronchus, were assessed *via* fully automated tree-like structure tracking. Vascular characteristics, such as the average diameter of vessels (AVD) at distances of 9, 15, and 21 mm from the pleura, the count of total vessels (N_total_), the count of small vessels with a cross-sectional area less than 5 mm^2^ (N_CSA_), and total blood volume (TBV), were extracted using multi-scale vessel enhancement filtering.

**Figure 2:**
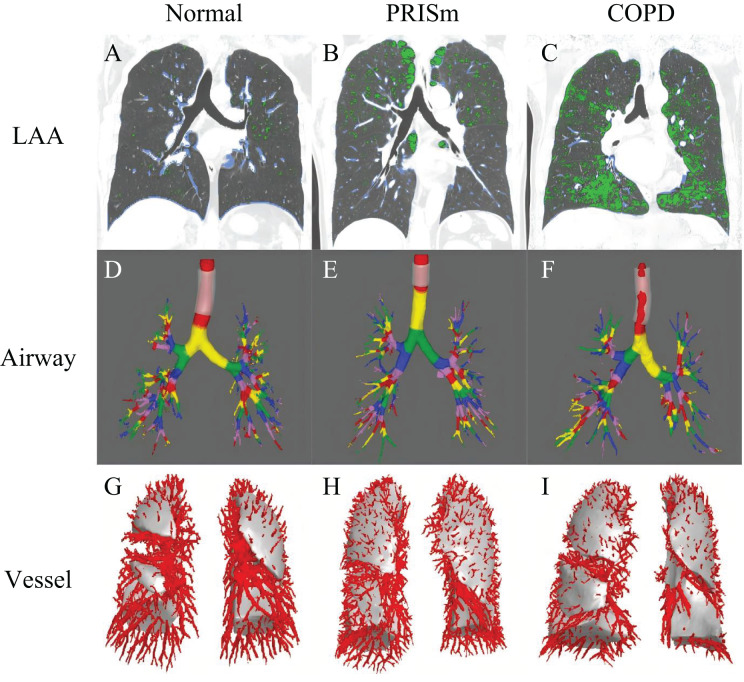
The framework for quantitative analysis of chest CT parameters. (A–C) Display green regions representing areas of low attenuation (LAA, ≤−950 HU) and blue regions indicating areas of high attenuation (HAA, −600 to −250 HU) in subjects with normal spirometry, PRISm and COPD. (D–F) Use different colors to denote the quantified airway grades for these three groups: normal spirometry, PRISm, and COPD. (G–I) Show the count of pulmonary vessels located 15 mm from the pleura in subjects categorized under normal spirometry, PRISm, and COPD. LAA areas of low attenuation, HAA areas of high attenuation, PRISm preserved ratio impaired spirometry, COPD chronic obstructive pulmonary disease.

To ensure accuracy, two senior radiologists (with 9 and 22 years of experience, respectively) conducted blinded reviews on 20% of randomly selected samples, achieving an intraclass correlation coefficient (ICC) greater than 0.85 (95% confidence interval (CI) [0.943–0.983]). Examples of quantitative CT parameters for the normal spirometry group, PRISm group, and COPD group are illustrated in Fig. 2.

### Statistical analysis

Statistical analyses were performed using Stata 23.0 (StataCorp LLC, College Station, TX, USA). One-way analysis of variance (ANOVA) was employed to compare clinical characteristics among the three groups. For data with unequal variances, Tamhane’s T2 test was utilized for intergroup comparisons. Spearman rank correlation analysis was conducted to evaluate the associations between quantitative CT parameters (such as Pi10, MLD, vessel diameter, *etc*.) and pulmonary function indicators (FEV1% predicted value, FEV1/FVC ratio, and MEF).

Four binary logistic regression models were constructed: ① PRISm *vs*. the normal group; ② PRISm *vs*. mild COPD (GOLD I & II); ③ PRISm *vs*. severe COPD (GOLD III & IV); and ④ PRISm *vs*. all stages of COPD (GOLD I–IV). Stepwise regression methods (entry criterion *P* < 0.2) were used to select predictive variables. The performance of these models was assessed through receiver operating characteristic (ROC) curves, with areas under the curve (AUC) and their 95% confidence intervals calculated. All statistical tests were two-sided, with a significance level set at *P* < 0.05. Data visualization was achieved using R 4.2, which can be downloaded from the official website http://www.R-project.org.

## Results

### Pulmonary function and whole-lung quantitative CT parameters

As shown in Table 1, there were significant gradient differences among the normal group (*n* = 135), PRISm group (*n* = 328), and COPD group (*n* = 195) regarding demographic characteristics, pulmonary function, and whole-lung CT parameters. Disease progression was associated with increasing age (64.07 ± 10.40 years for the normal group *vs*. 69.05 ± 9.93 years for the PRISm group *vs*. 73.28 ± 9.14 years for the COPD group, *P* < 0.001). Additionally, the proportion of males was significantly higher in the COPD group compared to the PRISm and normal groups (84.62% *vs*. 57.93% *vs*. 51.11%, respectively, *P* < 0.001). Although BMI and current smoking rates did not differ significantly between groups (*P* > 0.05), pulmonary function parameters such as FEV1% predicted value, peak expiratory flow (PEF), FEV1/FVC ratio, and mid-expiratory flow (MEF25-75) showed significant differences between the COPD group and the other two groups (*P* < 0.001). Similarly, whole-lung CT parameters including mean lung density (MLD), emphysema index (EI), and PI-1, PI-15 demonstrated significant variations across these groups (*P* < 0.001).

**Table 1:** Demographic, pulmonary function, and CT parameter comparisons (*n* = 658).

	Normal (*n* = 135)	PRISm (*n* = 328)	COPD (*n* = 195)	*P value*
Age (years)	64.07 ± 10.40	69.05 ± 9.93***	73.28 ± 9.14^###^	<0.001
Sex (male/female), *n*	69/66	190/138	165/30^###^	<0.001
Currently smoking (*n*, %)	27 (20)	74 (22.56)	56 (28.72)	0.139
BMI (kg/m^2^)	22.90 ± 4.65	22.51 ± 4.33	21.85 ± 4.58	0.091
**Pulmonary function parameters**				
FVC (L)	2.76 ± 0.71	2.1 ± 0.57***	2.38 ± 0.75^###^	<0.001
FEV1 (L)	2.41 ± 0.61	1.71 ± 0.45***	1.47 ± 0.56^###^	<0.001
FEV1% predicted (%)	96.98 ± 11.22	69.14 ± 9.08***	59 ± 19.25^###^	<0.001
FEV1/FVC (%)	0.87 ± 0.05	0.82 ± 0.07***	0.61 ± 0.08^###^	<0.001
PEF (L/s)	5.54 ± 1.76	4.13 ± 1.40***	3.31 ± 1.37^###^	<0.001
MEF75 (L/s)	5.35 ± 1.68	3.74 ± 1.26***	2.25 ± 1.19^###^	<0.001
MEF50 (L/s)	3.69 ± 1.12	2.14 ± 0.80***	1.01 ± 0.52^###^	<0.001
MEF25 (L/s)	1.31 ± 0.47	0.73 ± 0.37***	0.37 ± 0.22^###^	<0.001
DLco% predicted (%)	87.52 ± 19.54	70.8 ± 21.12***	63.04 ± 23.33^###^	<0.001
RV (L)	50.02 ± 8.31	53.14 ± 9.22***	57.69 ± 9.93^###^	<0.001
TLC (L)	4.89 ± 1.13	4.32 ± 1.10***	4.84 ± 1.08^###^	<0.001
**Whole lung parameters on CT**				
MLD (HU)	−781.37 ± 51.43	−779.43 ± 54.64	−828.93 ± 43.64^###^	<0.001
EI (%)	2.65 ± 4.47	2.71 ± 4.81	9.68 ± 10.48^###^	<0.001
PI-1 (HU)	−945.53 ± 40.23	−944.43 ± 44.08	−976.56 ± 31.79^###^	<0.001
PI-15 (HU)	−890.98 ± 35.56	−886.08 ± 41.20	−927.01 ± 32.56^###^	<0.001
Pi10 (mm)	3.26 ± 0.91	3.65 ± 0.89***	3.74 ± 0.83^#^	<0.001
TBV (cc)	174.76 ± 95.95	185.99 ± 54.31	215.21 ± 73^###^	<0.001

**Note:**

***P < 0.001, compared to the group of normal and PRISm, ^#^*P* < 0.05, ^###^*P* < 0.001, compared to the group of PRISm and COPD. A comparison of age, sex distribution, BMI, smoking status, pulmonary function indices (*e.g*., FEV1%, FEV1/FVC, PEF), and whole-lung CT metrics (*e.g*., MLD, EI%, Pi10) among Normal (*n* = 135), PRISm (*n* = 328), and COPD (*n* = 195) groups. Significant gradients emerged: COPD patients were older (73.3 *vs*. 69.1 *vs*. 64.1 years) and predominantly male (84.6% *vs*. 57.9% *vs*. 51.1%). Pulmonary function (FEV1%, FEV1/FVC) and CT parameters (Pi10: PRISm 3.65 mm *vs*. Normal 3.26 mm) worsened progressively (*P* < 0.001), reflecting structural-functional deterioration.

Notably, the parameter Pi10, which reflects small airway remodeling, showed a significant difference between the PRISm group (3.65 ± 0.89) and the normal group (3.26 ± 0.91) (*P* < 0.001).

### Regional specific pulmonary vascular parameters

Table 2 showed quantitative chest CT parameters in 658 participants (Normal: 135, PRISm: 328, COPD: 195), revealing heterogeneous vascular patterns at different distances from the pleural surface. Superficial region (9 mm): the PRISm group exhibited significantly smaller average vessel diameter (AVD_9_: 2.64 ± 0.38 mm) compared to the normal group (2.95 ± 0.86 mm, *P* < 0.001), approaching the COPD group (2.60 ± 0.53 mm). The count of small vessels with cross-sectional area <5 mm^2^ (N_CSA9_) was significantly higher in the COPD group (651.35 ± 398.31) than in the PRISm group (549.52 ± 326.3, *P* < 0.01). Local emphysematous regions (S_LAA9_) were markedly enlarged in the COPD group (42,269.16 ± 27,921.2 mm^2^, *P* < 0.001), while no differences were observed between PRISm and normal groups (*P* > 0.05). Intermediate region (15 mm): Vessel cross-sectional area (CSA_15_) in the PRISm group (4,000.06 ± 1,578.05 mm^2^) showed no statistical difference from the normal group (3,869.71 ± 2,400.55 mm^2^, *P* = 0.354) but was significantly lower than in the COPD group (4,442.73 ± 1,795.96 mm^2^, *P* < 0.01). Total vessel count (N_total15_) in the COPD group (686.59 ± 256.85) was significantly higher than in the PRISm group (544 ± 208.71, *P* < 0.01). Deep region (21 mm): the COPD group demonstrated a significantly increased vessel count (N_total21_: 385.02 ± 162.08) compared to the PRISm group (282.22 ± 139.26, *P* < 0.001). Local emphysematous areas (S_LAA21_) were consistently enlarged in the COPD group (17,148.32 ± 13,622.01 mm^2^, *P* < 0.001) across all depths.

**Table 2:** Comparison of CT-based quantitative parameters measured at 9, 15, and 21 mm from the pleural surface across the three groups (*n* = 658).

	Normal (*n* = 135)	PRISm (*n* = 328)	COPD (*n* = 195)	*P value*
**9 mm from the pleural surface**				
AVD_9_ (mm)	2.95 ± 0.86	2.64 ± 0.38***	2.60 ± 0.53	<0.001
CSA_9_ (mm^2^)	4,870.8 ± 3,056.66	5,195.21 ± 1,765.1	5,086.21 ± 2,164.41	0.354
N_total9_ (ea)	882.23 ± 607.48	909.6 ± 387.69	988.21 ± 503.56	0.088
N_CSA9_ (ea)	559.81 ± 442.6	549.52 ± 326.3	651.35 ± 398.31^##^	0.003
S_LAA9_ (mm^2^)	18,312.55 ± 17,214.71	18,111.6 ± 16,184.13	42,269.16 ± 27,921.2^###^	<0.001
**15 mm from the pleural surface**				
AVD_15_ (mm)	3.31 ± 0.64	3.04 ± 0.39***	2.94 ± 0.46^#^	<0.001
CSA_15_ (mm^2^)	3,869.71 ± 2,400.55	4,000.06 ± 1,578.05	4,442.73 ± 1,795.96^##^	0.007
N_total15_ (ea)	485.33 ± 309.82	544 ± 208.71*	686.59 ± 256.85^###^	<0.001
N_CSA15_ (ea)	215.37 ± 171.46	244.63 ± 140.86	334.22 ± 182.67^###^	<0.001
S_LAA15_ (mm^2^)	12,208.58 ± 12,004.37	11,446.97 ± 11,286.5	29,002.25 ± 20,410.79^###^	<0.001
**21 mm from the pleural surface**				
AVD_21_ (mm)	3.42 ± 0.61	3.24 ± 0.42**	3.16 ± 0.48	0.001
CSA_21_ (mm^2^)	2,195.4 ± 1,550.44	2,266.18 ± 1,254.16	2,985.36 ± 1,364.76^###^	<0.001
N_total21_ (ea)	252.1 ± 179.71	282.22 ± 139.26	385.02 ± 162.08^###^	<0.001
N_CSA21_ (ea)	103.57 ± 92.28	110.99 ± 72.47	162.92 ± 94.83^###^	<0.001
S_LAA21_ (mm^2^)	7,101.97 ± 7,837.48	6,257.22 ± 6,910.35	17,148.32 ± 13,622.01^###^	<0.001

**Note:**

*P < 0.05, **P < 0.01, ***P < 0.001, compared to the groups between normal and PRISm; ^#^*P* < 0.05, ^##^*P* < 0.01, ^###^*P* < 0.001, compared to the groups between PRISm and COPD. A comparison of vascular and emphysema metrics at 9, 15, and 21 mm depths from the pleura among Normal, PRISm, and COPD groups. PRISm showed reduced superficial vessel diameter (AVD_9_: 2.64 *vs*. 2.95 mm in Normal, *P* < 0.001), nearing COPD levels (2.60 mm). COPD exhibited higher small vessel counts (N_CSA9_: 651 *vs*. 550 in PRISm) and enlarged emphysema areas (S_LAA9_: 42,269 *vs*. PRISm/Normal, *P* < 0.001). Progressive vascular changes (*e.g*., N_total21_: COPD 385 *vs*. PRISm 282) highlight spatial heterogeneity, suggesting PRISm as a transitional state.

### CT imaging-pulmonary function correlation heatmap

**Figure 3:**
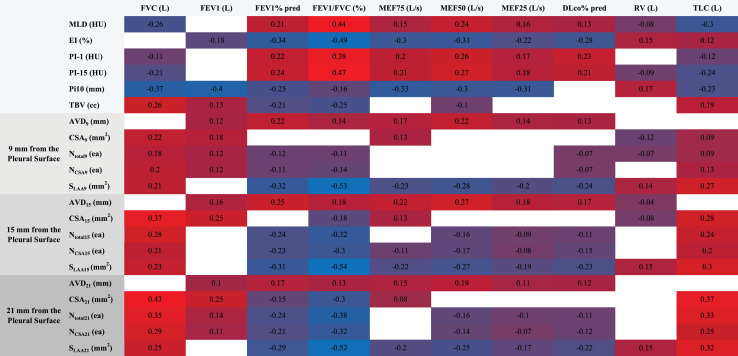
Heatmap of CT parameters and pulmonary function correlations. This heatmap visualizes Spearman correlations between quantitative CT parameters (*e.g*., MLD, EI%, Pi10, vessel metrics at 9/15/21 mm depths) and pulmonary function indices (FEV1%, FVC, FEV1/FVC). MLD negatively correlated with FVC and TLC (ρ = −0.26 to −0.30), while EI% showed strong negative associations with FEV1% (ρ = −0.34) and FEV1/FVC (ρ = −0.49). Pi10 correlated inversely with FEV1 (ρ = −0.40) and FVC (ρ = −0.37). Vascular parameters (*e.g*., superficial vessel diameter) demonstrated spatial heterogeneity, linking structural changes to functional decline in PRISm-to-COPD progression (*P* < 0.001).

Spearman correlation analysis revealed a multi-level association between quantitative CT parameters and pulmonary function indices (Fig. 3). Among lung parenchyma parameters, mean lung density (MLD) showed a negative correlation with forced vital capacity (FVC) and total lung capacity (TLC) (ρ = −0.26 for FVC, ρ = −0.30 for TLC, *P* < 0.001), whereas the emphysema index (EI%) was significantly negatively correlated with FEV1% predicted and FEV1/FVC ratio (ρ = −0.34 for FEV1%, ρ = −0.49 for FEV1/FVC, *P* < 0.001). The airway wall thickening marker Pi10 demonstrated strong negative correlations with FEV1, FVC, and mid-expiratory flow at 75% of vital capacity (MEF75) (ρ = −0.40 for FEV1, ρ = −0.37 for FVC, ρ = −0.33 for MEF75, *P* < 0.001).

Spatial heterogeneity in vascular parameters was pronounced: superficial layer (9 mm depth) surface low attenuation area (S_LAA_) exhibited a strong negative correlation with FEV1/FVC (ρ = −0.53, *P* < 0.001); intermediate layer (15 mm depth) cross-sectional area (CSA_15_) showed a negative correlation with diffusing capacity for carbon monoxide % predicted (DLCO%) (ρ = −0.11, *P* = 0.01), and total vessel count (N_total15_) had a negative correlation with residual volume to total lung capacity ratio (RV/TLC) (ρ = −0.24, *P* < 0.001); deep layer (21 mm depth) small vessel number (N_CSA21_) was negatively correlated with FEV1/FVC (ρ = −0.32, *P* < 0.001), suggesting a gradient effect of microvascular disease. Total blood volume (TBV) was negatively correlated with FEV1% predicted (ρ = −0.21) but positively correlated with TLC (ρ = 0.19, both *P* < 0.001).

### Validation of diagnostic model performance

**Figure 4:**
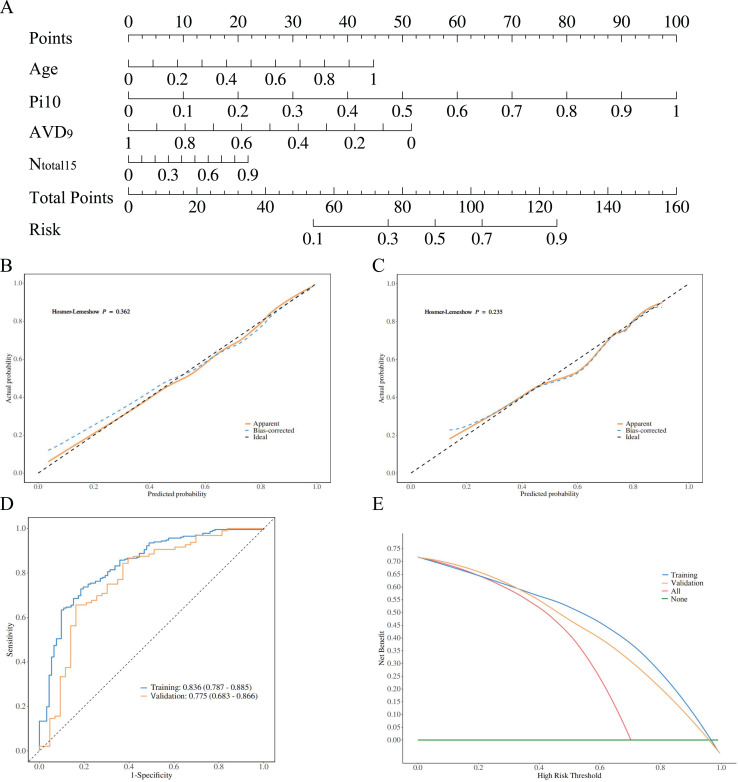
Model performance visualization. Radiomic features, calibration curves, ROC curves, and decision curve analysis (DCA). (A) Highlights key predictors (age, Pi10, AVD_9_, Ntotal_15_). Calibration curves (B, C) Show good model fit (Hosmer-Lemeshow *P* > 0.05). ROC curves (D) Demonstrate strong discrimination (training AUC: 0.836; validation: 0.775). DCA (E) Indicates clinical utility when threshold probability exceeds 20%. Collectively, the model achieves high accuracy and applicability for PRISm *vs*. normal differentiation.

**Figure 5:**
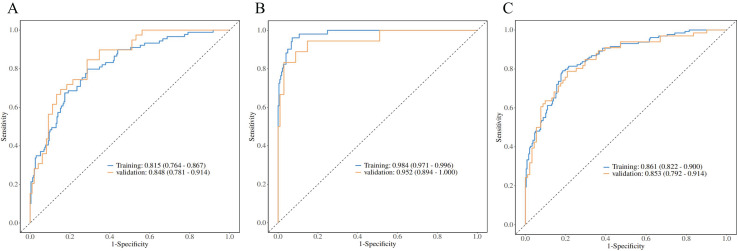
PRISm *vs*. COPD logistic regression analysis. ROC curves comparing PRISm with mild (A), severe (B), and all COPD stages (C). Models achieved high AUCs: 0.815 (training)/0.853 (validation) for mild COPD, 0.984/0.952 for severe COPD, and 0.861/0.853 for all stages. Hosmer-Lemeshow tests confirmed calibration (*P* > 0.05). The gradient in performance (AUC: severe > mild > all) underscores the model’s precision in distinguishing early *vs*. advanced COPD, supporting clinical stratification.

The diagnostic models constructed using stepwise logistic regression across four groups demonstrated a gradient of discriminative performance (Figs. 4–5). For distinguishing between PRISm and normal groups, the model incorporated age, normalized airway wall thickness (Pi10), average vessel diameter at 9 mm depth (AVD_9_), and total vessel count (N_total15_). The predictive probability formula is defined as *P* = e*x*/(1 + e*x*), *x* = 0.961 + 1.929 * Age + 6.373 * Pi10−8.673 * AVD_9_ + 8.179 * N_total15_. The nomogram converts these parameters into a visual scoring system where each predictor contributes points based on its value (Fig. 4A). To use the nomogram clinically: (1) locate each patient’s value on the corresponding axis, (2) draw a vertical line to the ‘Points’ axis to obtain individual scores, (3) sum all points to get ‘Total Points’, and (4) draw a vertical line from ‘Total Points’ to the ‘Risk’ axis to obtain the probability of PRISm. The optimal cutoff probability is 0.25 (sensitivity: 71.3%, specificity: 79.8%), meaning patients with predicted probability >0.25 should be classified as PRISm. For example, a 70-year-old patient with Pi10 = 3.8 mm, AVD_9_ = 2.5 mm, and N_total15_ = 600 would accumulate approximately 95 total points, corresponding to a 65% PRISm probability, indicating high-risk classification. This model achieved an AUC of 0.836 (95% CI [0.787–0.885]) in the training set and 0.775 (95% CI [0.683–0.866]) in the validation set (Fig. 4D). The Hosmer-Lemeshow test indicated good calibration performance for the model (training set χ^2^ = 8.776, *P* = 0.362; validation set χ^2^ = 10.447, *P* = 0.235) (Figs. 4B, 4C). Decision curve analysis (DCA) further confirmed that when the threshold probability exceeded 20%, the model provided significant clinical net benefit both in the training set and validation set (Fig. 4E).

For the differentiation between PRISm and COPD subgroups, all models passed the Hosmer-Lemeshow goodness-of-fit test (*P* > 0.05), demonstrating a disease severity-dependent gradient in model performance (Table 3, Fig. 5). Specifically: PRISm *vs*. mild COPD (GOLD I & II): models utilizing the total number of vessels in the subpleural regions at 15 mm/21 mm depths (N_total15_ and N_total21_) and the count of small vessels (N_CSA21_) as core predictors achieved AUCs of 0.815 (95% CI [0.764–0.867]) in the training set and 0.853 (95% CI [0.792–0.914]) in the validation set. PRISm *vs*. Severe COPD (GOLD III & IV): The model integrating Age, normalized airway wall thickness (Pi10), and total blood volume (TBV) performed optimally, with an AUC of 0.984 (95% CI [0.971–0.996]) in the training set and 0.952 (95% CI [0.894–1.000]) in the validation set, achieving a specificity of 98.7%. PRISm *vs*. All COPD (GOLD I–IV): utilizing parameters such as PI-15, cross-sectional area of vessels at 15 mm depth (CSA_15_), and localized emphysema (S_LAA15_), the model attained AUCs of 0.861 (95% CI [0.822–0.900]) in the training set and 0.853 (95% CI [0.792–0.914]) in the validation set.

**Table 3:** Binary logistic regression results for PRISm *vs*. COPD groups.

Groups	Data	Cut-off	AUC (95% CI)	Accuracy (95% CI)	Sensitivity (95% CI)	Specificity (95% CI)	H-L test	*P value*
PRISm *vs*. Mild COPD	Training	0.251	0.815 [0.764–0.867]	0.737 [0.685–0.784]	0.713 [0.655–0.772]	0.798 [0.714–0.881]	6.861	0.552
	Validation		0.848 [0.781–0.914]	0.745 [0.663–0.815]	0.714 [0.625–0.804]	0.821 [0.700–0.941]	3.995	0.858
PRISm *vs*. Severe COPD	Training	0.238	0.984 [0.971–0.996]	0.935 [0.899–0.961]	0.929 [0.896–0.963]	0.961 [0.908–1.000]	10.340	0.242
	Validation		0.952 [0.894–1.000]	0.900 [0.832–0.947]	0.912 [0.857–0.967]	0.833 [0.661–1.000]	3.261	0.917
PRISm *vs*. All COPD	Training	0.374	0.861 [0.822–0.900]	0.806 [0.762–0.845]	0.814 [0.765–0.864]	0.791 [0.720–0.861]	12.425	0.133
	Validation		0.853 [0.792–0.914]	0.771 [0.697–0.834]	0.835 [0.759–0.911]	0.682 [0.569–0.794]	3.771	0.877

**Note:**

This table summarizes binary logistic regression models distinguishing PRISm from mild (GOLD I–II), severe (GOLD III–IV), and all COPD stages. Key predictors included vascular parameters (*e.g*., N_total15_, TBV), Pi10, and age. Models achieved high AUCs: 0.815 (training) and 0.853 (validation) for PRISm *vs*. mild COPD; 0.984 (training) and 0.952 (validation) for PRISm *vs*. severe COPD. All models showed excellent calibration (Hosmer-Lemeshow *P* > 0.05), validating robustness for early COPD stratification.

## Discussion

### Radiological evidence of PRISm as a precursor stage of COPD

Our study identified significant differences between the PRISm group and the normal group in terms of airway wall thickening (Pi10: 3.65 ± 0.89 *vs*. 3.26 ± 0.91 in the normal group, *P* < 0.001) and reduced superficial vessel diameter at 9 mm from the pleura (2.64 ± 0.38 mm *vs*. 2.95 ± 0.86 mm in the normal group, *P* < 0.001). However, there was no difference in the emphysema index (LAA-950) between the two groups. These findings align with those of the Framingham cohort study (Synn et al., 2019), which indicated that small airway disease increases before the onset of emphysema in PRISm, supporting its role as a “silent precursor phase” to COPD (Wan et al., 2018).

The pathological research by Agustí & Hogg (2019) further corroborates this by demonstrating that narrowing or disappearance of terminal bronchioles is a core event in the early stages of COPD. Additionally, related dual-phase CT studies have validated this mechanism at the imaging level, showing an increase in PRM^fSAD^ in the PRISm group compared to the normal group (21.7% *vs*. 15.4%, *P* < 0.01) (Lu et al., 2022).

However, it is noteworthy that our study found no significant differences in subpleural low attenuation areas (S_LAA_) at 9, 15, and 21 mm from the pleura between the PRISm and normal groups (*P* > 0.05). This could be associated with peribronchiolar fibrosis or compensatory thickening of the alveolar septa, suggesting that early lung structural changes are not merely destructive processes but may involve reparative mechanisms as well (Bhatt et al., 2019). In summary, these studies underscore the paramount importance of quantitative CT in diagnosing and stratifying PRISm, particularly concerning parenchymal and small airway alterations. These provide innovative insights for subsequent clinical research and therapeutic interventions.

### Clinical advantages and challenges of single-inspiratory phase CT

Traditional dual-phase CT is limited by radiation dose constraints, hindering its widespread adoption. Additionally, deep learning models are often criticized for their “black box” nature, which complicates clinical decision-making. This study innovatively uses a single inspiratory-phase CT scan combined with a logistic regression model to efficiently differentiate between PRISm and individuals with normal spirometry or mild-to-moderate COPD (AUC = 0.836 for PRISm *vs*. normal spirometry; AUC = 0.815 for PRISm *vs*. mild-to-moderate COPD), while maintaining parameter interpretability. This approach aligns with the concept of “one-stop CT screening” proposed by Zhou et al. (2023), which leverages standard inspiratory-phase images from lung cancer screening CT scans to simultaneously assess COPD risk, thereby achieving multiple assessments in one examination.

For instance, Pi10 serves as a core predictive factor in our model and can directly guide clinical interventions such as smoking cessation reinforcement or anti-inflammatory treatment. According to the 2023 GOLD guidelines, PRISm patients should be included in chronic disease management, encompassing regular pulmonary function monitoring, chest CT screening, and personalized interventions like bronchodilator use. Our nomogram model provides radiological support for these management strategies by identifying early markers such as reduced superficial small vessel counts (Ncsa < 5) and airway wall thickening (Pi10), enabling precise stratification of high-risk populations. Moreover, integrating real-world data into predictive modeling to forecast the risk of α−1 antitrypsin (AAT) deficiency and applying timely pharmacological interventions could further delay the progression from PRISm to COPD (Pfeffer et al., 2025).

However, single inspiratory-phase CT lacks dynamic expiratory phase imaging data (*e.g*., air trapping index, degree of airway collapse), which are crucial functional parameters. Consequently, it may fail to accurately capture airway deformation characteristics during the respiratory cycle and dynamic changes in gas distribution, limiting the assessment of reversible airway obstruction and regional ventilation heterogeneity (Galbán et al., 2012). Chen et al. (2023) introduced a deep learning model based on generative adversarial networks and UNet that generates high-quality expiratory phase CT scans from single inspiratory-phase CT images (Structural Similarity Index Measure (SSIM) = 0.86) and predicts small airway disease parameters such as Parameter Response Mapping (PRM). The predicted functional small airway disease (fSAD) volume percentage showed a strong correlation with actual values (r = 0.97) and effectively stratified small airway disease risk (AUC = 0.84). Future research could combine deep learning techniques to enhance model sensitivity further.

### Spatial heterogeneity of vascular parameters and pathological implications

Previous pathophysiological studies on COPD have demonstrated that chronic hypoxia, persistent fibroinflammatory responses, and multifactorial pathogenic stimuli impair pulmonary vascular endothelial growth factor (VEGF) function, leading to characteristic vascular remodeling. This process involves progressive volume atrophy of distal small vessels (vascular pruning) due to apoptosis and degeneration, alongside compensatory volume expansion of proximal vessels *via* wall thickening and lumen dilation. Such spatially heterogeneous dynamic remodeling is defined as the “vascular pruning and remodeling” phenomenon (Hueper et al., 2015; Rahaghi et al., 2016). Huang et al. (2022) applied the PRM method and found that emphysema volume percentage (Emph%) had a more significant impact on expiratory intrapulmonary vascular volume (IPVV) than functional small airway disease (fSAD%) (*P* < 0.05), highlighting its diagnostic value for emphysema-dominant vascular remodeling. However, this method exhibits limited sensitivity in PRISm and early-stage COPD patients due to the absence of significant emphysema.

This study systematically reveals early vascular pruning features in PRISm. Superficial region (9 mm): the PRISm group showed significantly reduced vessel diameter (Δ = 0.31 mm, *P* < 0.001), approaching the COPD group (2.60 ± 0.53 mm *vs*. PRISm: 2.64 ± 0.38 mm), indicating that distal vascular pruning (apoptosis and degeneration) precedes emphysema formation, consistent with early-stage distal atrophy in the “vascular pruning-remodeling” theory. This alteration is detectable in individuals with normal or mildly impaired spirometry, suggesting its role as a sensitive imaging biomarker for early COPD screening. Superficial vessel diameter (*P* < 0.001) outperformed traditional spirometric indices (*e.g*., FEV1%) in distinguishing PRISm from normal groups, particularly in emphysema-free stages. Compensatory increases in small vessel count (N_CSA_) in intermediate regions (COPD: 651.35 ± 398.31 *vs*. PRISm: 549.52 ± 326.3, *P* = 0.003) highlight spatial heterogeneity in remodeling: distal pruning coexists dynamically with proximal compensatory expansion.

Vascular changes in PRISm may represent a transitional state in the COPD continuum, with reduced superficial diameter serving as a predictive biomarker for disease progression. Integrating superficial vessel diameter with local emphysema metrics (*e.g*., S_LAA_) enhances the detection of early emphysema-vascular coupling, supporting the hypothesis that “superficial microvascular pruning precedes deep vascular rarefaction,” as aligned with Matsuoka et al. (2010). This phenomenon may relate to hypoxia-driven remodeling: early hypoxia induces superficial vasoconstriction, while emphysema progression exacerbates deep vascular loss *via* alveolar-capillary destruction (Lynch et al., 2015). Notably, although total blood vessel volume (TBV) in PRISm did not differ from normal groups, reduced small vessel density (N_CSA_ < 5: PRISm 549.52 ± 326.3 *vs*. COPD 651.35 ± 398.31, *P* = 0.003) reflects microcirculatory dysfunction preceding macroscopic vascular changes. This aligns with the “vascular pruning hypothesis” proposed by Washko et al. (2019), where vascular rarefaction correlates with poor COPD prognosis. This study is the first to identify characteristic superficial vessel diameter reduction (Δ = 0.31 mm) in PRISm, underscoring its potential as a sensitive marker for disease progression. These findings deepen the understanding of spatiotemporal heterogeneity in “vascular pruning-remodeling” and provide imaging-based evidence for early COPD stratification.

Additionally, our study successfully employed a single-inspiratory quantitative CT-based nomogram model to differentiate PRISm from various COPD subgroups. All models demonstrated excellent calibration, confirmed by the Hosmer-Lemeshow test (*P* > 0.05), and exhibited performance gradients associated with disease severity. The use of single-inspiratory phase CT parameters not only enhances diagnostic accuracy but also provides deeper insights into the transition from PRISm to COPD. The ability to detect subtle changes in small vessels and airways before the onset of overt emphysema supports the concept of “early warning” for disease progression. This early detection capability enables timely clinical interventions aimed at slowing or preventing the progression from PRISm to more severe forms of COPD. Specifically, identifying these early-stage biomarkers offers a critical window for therapeutic intervention, potentially altering the course of the disease.

In summary, this study demonstrates the potential of quantitative CT parameters obtained from a single inspiratory phase to construct highly accurate and interpretable models for distinguishing PRISm from different stages of COPD. By focusing on specific biomarkers such as vessel counts and airway dimensions, these models provide valuable tools for early detection and risk stratification. The findings underscore the importance of incorporating advanced imaging techniques into clinical practice, offering new avenues for personalized management strategies. Future research should aim to validate these results in larger and more diverse populations, further refining our understanding of disease progression and enhancing patient outcomes.

The core innovation of this study lies in the pioneering integration of spatially heterogeneous vascular parameters (*e.g*., vessel diameter and density at 9/15/21 mm from the pleural surface), transcending conventional lung parenchyma analysis frameworks to uncover transitional imaging features in PRISm populations. Furthermore, the adoption of an interpretable logistic regression model as an alternative to “black-box” deep learning significantly enhances clinical decision-making transparency. However, several limitations warrant acknowledgment: single-center retrospective design: potential selection bias inherent to this design necessitates external validation across multicenter cohorts (*e.g*., COPDGene) to confirm model generalizability (Dai et al., 2023). Additionally, while we demonstrated good inter-reader reliability (ICC > 0.85) through blinded review of 20% randomly selected samples by two senior radiologists, having both radiologists independently assess all cases would further strengthen measurement validity and enable comprehensive agreement analysis. Future multicenter studies should implement dual independent readings for all quantitative CT parameters to minimize measurement bias and establish robust quality control standards. Respiratory effort variability: differences in inspiratory effort may compromise the stability of quantitative CT (QCT) parameters. Future studies should integrate AI-driven image registration systems to standardize protocols. Limited multi-omic data: the exclusion of genomic or proteomic datasets restricts mechanistic exploration of PRISm heterogeneity, which could be addressed through integrated multi-omics approaches in subsequent research.

## Conclusions

This study redefines the imaging boundaries between PRISm and COPD through single-inspiratory quantitative CT parameters, highlighting the critical role of small airway remodeling and vessel diameter at 9 mm from the pleural surface as early biomarkers. The constructed nomogram model provides a reliable tool for precise identification of PRISm and individualized intervention, while offering novel insights into the pathophysiological mechanisms of COPD progression. Future interdisciplinary collaborations are warranted to facilitate the clinical translation of these imaging biomarkers into routine practice.

## Supplemental Information

10.7717/peerj.20575/supp-1 Supplemental Information 1 R code.

10.7717/peerj.20575/supp-2 Supplemental Information 2 Raw data.

10.7717/peerj.20575/supp-3 Supplemental Information 3 STROBE checklist.

## Additional Information and Declarations

### Data Availability

The following information was supplied regarding data availability:

The raw data is available in the Supplemental File.

### Clinical Trial Registration

The following information was supplied regarding Clinical Trial registration:
